# Hydrophilic Fungi and Ergosterol Associated with Respiratory Illness in a Water-Damaged Building

**DOI:** 10.1289/ehp.10355

**Published:** 2007-10-09

**Authors:** Ju-Hyeong Park, Jean M. Cox-Ganser, Kathleen Kreiss, Sandra K. White, Carol Y. Rao

**Affiliations:** Division of Respiratory Disease Studies, National Institute for Occupational Safety and Health, Morgantown, West Virginia, USA

**Keywords:** asthma, dampness, endotoxin, ergosterol, exposure, hydrophilic fungi, office building, respiratory symptoms, water damage

## Abstract

**Background:**

Damp building–related respiratory illnesses are an important public health issue.

**Objective:**

We compared three respiratory case groups defined by questionnaire responses [200 respiratory cases, 123 of the respiratory cases who met the epidemiologic asthma definition, and 49 of the epidemiologic asthma cases who had current physician-diagnosed asthma with post-occupancy onset] to a comparison group of 152 asymptomatic employees in an office building with a history of water damage.

**Methods:**

We analyzed dust samples collected from floors and chairs of 323 cases and comparisons for culturable fungi, ergosterol, endotoxin, and cat and dog allergens. We examined associations of total fungi, hydrophilic fungi (requiring water activity ≥ 0.9), and ergosterol with the health outcomes using logistic regression models.

**Results:**

In models adjusted for demographics, respiratory illnesses showed significant linear exposure–response relationships to total culturable fungi [interquartile range odds ratios (IQR-OR) = 1.37–1.72], hydrophilic fungi (IQR-OR = 1.45–2.19), and ergosterol (IQR-OR = 1.54–1.60) in floor and chair dusts. Of three outcomes analyzed, current asthma with postoccupancy physician diagnosis was most strongly associated with exposure to hydrophilic fungi in models adjusted for ergosterol, endotoxin, and demographics (IQR-OR = 2.09 for floor and 1.79 for chair dusts). Ergosterol levels in floor dust were significantly associated with epidemiologic asthma independent of culturable fungi (IQR-OR = 1.54–1.55).

**Conclusions:**

Our findings extend the 2004 conclusions of the Institute of Medicine [Human health effects associated with damp indoor environments. In: Damp Indoor Spaces and Health. Washington DC:National Academies Press, 183–269] by showing that mold levels in dust were associated with new-onset asthma in this damp indoor environment. Hydrophilic fungi and ergosterol as measures of fungal biomass may have promise as markers of risk of building-related respiratory diseases in damp indoor environments.

Mold is ubiquitous in normal indoor and outdoor environments; thus, some exposure is inevitable in everyday life. However, exposure to increased levels of mold and other microbial agents has been implicated in diseases associated with damp indoor environments (Menzies and Kreiss 2006; Park et al. 2006). In the absence of indoor amplification, the fungal profiles inside buildings should be similar to those outdoors (Flannigan and Miller 2001). Increased moisture levels due to water intrusion can support mold growth and may change the profile of fungal populations in a building. Hydrophilic (water-loving) fungi requiring ≥ 0.9 water activity ( *A_w_* ; the amount of free or available water in substrates) will overgrow mesophilic (0.8 ≤ *A_w_* < 0.9) and xerophilic fungi ( *A_w_* < 0.8) in damp conditions (Flannigan and Miller 2001; Grant et al. 1989). The presence of hydrophilic fungi is considered an indicator of building dampness (Flannigan and Miller 2001), yet quantitative measures of hydrophilic fungi in damp buildings have not had been studied in relation to health effects.

The analytical method most frequently applied for fungi, a culture technique, is not likely to measure the relevant microbial exposures accurately, because any selected medium grows only a small proportion of the viable spores and because culture counts do not account for nonviable spores and fungal fragments. However, ergosterol, a principal sterol in the fungal membrane, has been suggested as a good measure for fungal biomass (Newell 1994; Sebastian and Larsson 2003; Szponar and Larsson 2000) since it is analyzed by a chemical technique which measures viable and nonviable spores and fungal fragments. However, only a few researchers have attempted to measure ergosterol in environmental samples for assessing exposure to fungi in epidemiologic studies (Dales 1998; Dales et al. 1999; Dharmage et al. 2001, 2002; Matheson et al. 2005; Mendell et al. 2002). More research using ergosterol measurements for fungal exposure assessment would be useful to better understand the association of fungal exposure with health.

In this study we focused on examining associations of hydrophilic fungi and ergosterol with respiratory health outcomes among employees in a 20-story office building in the northeastern United States. Within a few months of building occupancy in 1994, employees perceived new-onset respiratory conditions to be building-related and complained of a further increase in symptom severity and frequency beginning in the fall of 2000. Sentinel cases of postoccupancy-onset asthma, hypersensitivity pneumonitis (HP), and sarcoidosis had been diagnosed. A building-wide self-administered questionnaire survey in September 2001 (67% participation rate; 888 of 1,327) documented an excess of respiratory symptoms and asthma prevalence, and a 7.5-fold increased incidence density of adult posthire-onset asthma (Cox-Ganser et al. 2005; Park et al. 2006) compared with preoccupancy incidence.

## Materials and Methods

### Case and comparison group definitions

We nested a case–comparison study within the participants in the 2001 cross-sectional questionnaire survey. We defined respiratory cases (*n* = 200) as those who had occupied the building at least 1 year and reported *a*) current asthma with postoccupancy physician diagnosis, physician-diagnosed HP, or sarcoidosis (potential building-related respiratory disease diagnoses); or *b*) three or more of five asthma-like symptoms (wheeze or whistling in the chest, chest tightness, shortness of breath, coughing, and awakened by breathing difficulty) occurring weekly over the past 4 weeks; or *c*) two or more of three symptoms consistent with HP (shortness of breath when hurrying on level ground or walking up a slight hill, fever and chills, or flu-like achiness or achy joints) occurring weekly over the past 4 weeks. Because we were interested in restricting analyses to asthma as the outcome, we defined epidemiologic asthma (epi-asthma) cases (*n* = 123) as having current asthma with post-occupancy physician diagnosis, or three or more of five asthma-like symptoms. Finally, to increase the specificity of our asthma definition, we defined postoccupancy asthma cases (*n* = 49) as those with current asthma with postoccupancy physician diagnosis. The epi-asthma cases were a subset of the respiratory cases, and the postoccupancy asthma cases were a subset of the epi-asthma cases. We defined the comparison group (*n* = 152) as those who reported none of the lower respiratory or HP-like symptoms in the past year, as well as no physician-diagnosed HP, sarcoidosis, or postoccupancy asthma. An informed consent procedure, approved by the National Institute for Occupational Safety and Health Human Subjects Review Board, was followed before participants completed the questionnaire.

### Environmental sampling and analysis

In April 2002, we collected floor and chair dust samples from workstations of 323 case and comparison group employees. We sent dust samples for analyses of culturable fungi, ergosterol, endotoxin, cat allergen, and dog allergen. Fungal colonies were cultured with malt extract (selective for a broad spectrum of fungi), cellulose (selective for cellulolytic fungi), and dichloran 18% glycerol (selective for xerophilic fungi) agars at room temperature for 7–10 days, identified to species level, and enumerated. If a species grew on more than one medium, a standardized laboratory protocol was used to select which medium would be the basis for the reported colony count results. Ergosterol was analyzed with gas chromatography-mass spectrometry (Sebastian and Larsson 2003). Endotoxin was analyzed using the kinetic quantitative chromogenic Limulus amoebocyte lysate (KQCL) method (Chun et al. 2002). Cat (Fel d 1) and dog (Can f 1) allergens were analyzed with an enzyme-linked immunosorbent assay (Chapman 1988; de Groot 1991). For epidemiologic analyses, we used units per square meter or per chair based on our previous findings (Park et al. 2006). Details of the methods are described elsewhere (Park et al. 2006).

### Data analysis

Because of right-skewed distributions, we transformed all the environmental measurements using natural logarithms and reported geometric means (GMs) and geometric SDs (GSDs) by exponentiating the means and SDs of the log-transformed data. We assigned a value of half the limit of detection (LOD) to samples < LOD because of the large GSD (Hornung and Reed 1990). We grouped fungal species into mesophilic (0.8 ≤ *A_w_* < 0.9) and hydrophilic (*A_w_* ≥ 0.9) categories based on *A_w_* (Burge and Otten 1999; Flannigan and Miller 2001; Grant et al. 1989) and created a combined group of mesophilic and hydrophilic fungi (*A_w_* ≥ 0.8). We created two additional groups: fungi not classified as having an *A_w_* ≥ 0.8 and fungi not classified as hydrophilic.

We used analysis of variance to compare the levels of microbial agents in floor and chair dust. We estimated odds ratios (ORs) for each case definition in relation to various microbial indices using multivariate linear logistic regression models (SAS 9.1; SAS Institute Inc., Cary, NC). Single environmental variable models included one environmental variable and demographics (age, sex, race, smoking status, and building occupancy time), which are potential confounding factors; we also adjusted for these variables in the models used in our previous study (Park et al. 2006). Multiple environmental variable models included demographics and three environmental variables [ergosterol, endotoxin, and total fungi (total fungi models) or hydrophilic fungi (hydrophilic fungi models)]. We performed additional analyses to examine the effects of fungi that were not classified as having an *A_w_* ≥ 0.8 or those not classified as hydrophilic on health outcomes using the single environmental variable models. Because the interactions among these environmental variables were not significant for all three outcomes, we did not include interactions in the final models. We examined possible nonlinear relationships between exposure and health outcomes using generalized additive models (GAMs) with a smoothing spline function (degrees of freedom = 4) (S-Plus 6.1; Insightful Corporation, Seattle, WA). We reported adjusted ORs and 95% confidence intervals (CIs) based on increase in exposure by interquartile range (IQR = 75th percentile – 25th percentile).

## Results

On average, the cases and comparisons in the study were 46 years of age and had occupied the building for 6 years (Table 1). More than half of them were white (69%), never-smokers (61%), and female (59%). There were fewer white employees and never-smokers but more females in the case groups than in the comparison group. The proportion of current smokers was lowest (6.1%) in the postoccupancy asthma cases.

We collected 338 floor and 327 chair dust samples from 323 employees’ workstations among the 352 case and comparison group employees. We could not locate workstations for 29 participants. For those who had multiple samples because of changes in their workstations between September 2001 and April 2002, we assigned measurements of microbial agents from the workstation they occupied during the 2001 questionnaire survey. Because of the limited amount of dust collected for some samples, we prioritized sample analysis by endotoxin, culturable fungi, ergosterol, and allergens. We recovered a total of 67 fungal species from floor dust samples and 69 species from chair dust samples. In addition, unidentifiable species of *Penicillium*, yeasts (*Rhodotorula* and *Sporobolomyces*), and nonsporulating fungi were cultured. The GM of total culturable fungi was 7,700 colony-forming units (CFU) per gram in floor dust, which was significantly (*p* < 0.005) lower than that (11,000 CFU/g) in chair dust (Table 2). In the floor dust, on average, 57% of total fungal colonies were identified as hydrophilic fungi and 19% as mesophilic. In the chair dust, on average, 45% of total fungal colonies were identified as hydrophilic and 28% as mesophilic. Eighty-seven percent of the hydrophilic fungi in floor dust and 74% of those in chair dust were yeasts. GMs of the ergosterol (0.5 ng/mg) and endotoxin (10.9 EU/mg) levels in floor dust were significantly (*p*-values < 0.002) higher than those in chair dust (0.4 ng/mg and 2.1 EU/mg, respectively). The levels of cat (GM, 2.5 μg/g) and dog (2.1 μg/g) allergens were significantly (*p*-values < 0.0001) lower in floor dust than in chair dust (GMs, 12.5 μg/g and 5.7 μg/g, respectively) (Table 2).

*Aureobasidium pullulans* and *Epicoccum nigrum* were the most prevalent of the mesophilic fungi (Table 3). Yeasts and *Phoma herbarum* were the most prevalent hydrophilic fungi recovered, and their median concentrations (2,400 CFU/g in floor dust and 2,340 CFU/g in chair dust for yeasts; 2,850 CFU/g in floor dust and 3,600 CFU/g in chair dust for *Phoma herbarum*) were among the highest found for the mesophilic and hydrophilic fungi. *Rhodotorula* was the predominant genus of yeast identified from floor (49.4%) and chair (46.0%) dust samples. Among those that were not classified as having an *A_w_* of ≥ 0.8, *Penicillium chrysogenum* (12.5%) and *Pithomyces chartarum* (10.4%) were the most frequently found species in floor dust samples, with *Pithomyces chartarum* (23.3%) and *Phoma glomerata* (11.7%) most frequently in chair dust samples. *Penicillium chrysogenum* and *Aspergillus niger* were the most prevalent *Penicillium/Aspergillus* species identified in both floor and chair dust. Nonidentifiable *Penicillium* species were found in 17.1% and 13.5% of the floor and chair dust samples, respectively. We found *Stachybotrys chartarum* in four floor samples and five chair samples.

The levels of ergosterol per square meter or chair were significantly (*p*-values < 0.0001) but poorly correlated with total fungi, fungi requiring *A_w_* ≥ 0.8, hydrophilic fungi, and yeasts both in floor and chair dust samples (*r* = 0.15–0.27). Ergosterol had a higher correlation with endotoxin in floor dust (*r* = 0.47) and chair dust (*r* = 0.34). Ergosterol and culturable fungi were also significantly but weakly correlated with cat and dog allergens in both floor and chair dust (*r* = 0.12–0.31). Endotoxin had a higher correlation with cat (*r* = 0.39) and dog allergens (*r* = 0.36) in chair dust than in floor dust (*r* = 0.25 for cat and 0.21 for dog allergens).

The number of subjects in each of the statistical models varied, depending on the number of subjects with information missing in any of the demographic variables or the exposure variables. We found significant linear exposure–response relationships between various microbial measurements (total fungi, fungi requiring *A_w_* ≥ 0.8, hydrophilic fungi, ergosterol, and endotoxin) in dust and health outcomes (respiratory cases, epi-asthma cases, and postoccupancy asthma cases) in the single environmental variable models. We found no significant associations (at α = 0.05) among health outcomes and fungi that were not classified as having an *A_w_* ≥ 0.8, or fungi that were not classified as hydrophilic (Table 4). In GAMs, test *p*-values of these environmental variables for a null hypothesis of linearity were > 0.05, which indicates no evidence of nonlinear relationships between exposure and health outcomes. The associations between health outcomes and fungi were mostly driven by exposure to fungi requiring *A_w_* ≥ 0.8, and specifically hydrophilic fungi in both floor and chair dust.

In single environmental variable models, we found > 65% increases in the odds of being a respiratory case for increasing IQR exposure [IQR-OR = 1.66–1.73; *p*-values < 0.05] for total fungi, fungi requiring *A_w_* ≥ 0.8, and hydrophilic fungi in floor dust (IQR for each of the microbial measurements for all dust samples analyzed are presented in Table 2). Yeasts in chair dust and non-yeast hydrophilic fungi in floor dust were significantly associated with increases in the odds of being a respiratory case of 46% and 50%, respectively (Table 4). Ergosterol and endotoxin in floor dust were associated with significantly increased odds of being a respiratory case (IQR-OR = 1.56 and 1.60, respectively). Cat and dog allergens in floor dust were not significantly associated with respiratory cases at α = 0.05. In general, the odds of being a respiratory case for chair dust were lower than those for floor dust, except for the yeasts.

When compared to respiratory case outcome models, we found slightly larger magnitudes of IQR-ORs (range, 1.46–1.80) for epi-asthma cases associated with total culturable fungi, fungi requiring *A_w_* ≥ 0.8, hydrophilic fungi, and ergosterol in both floor and chair dust (Table 4). When we examined the associations of those who reported physician-diagnosed HP or having two or more HP-like symptoms with microbial exposures, we found similar magnitudes of ORs for fungi but stronger associations with ergosterol (IQR-OR = 1.93) and endotoxin (IQR-OR = 1.80) in floor dust, and yeasts in chair dust (IQR-OR = 1.65) (data not shown).

In the models with postoccupancy asthma cases as an outcome, the associations with fungi requiring *A_w_* ≥ 0.8, hydrophilic fungi, and yeasts in both floor and chair dust were stronger than those in the models with respiratory and epi-asthma cases as outcomes (Table 4). Of all the environmental variables, hydrophilic fungi in floor dust (IQR-OR = 2.19) were most strongly associated with post-occupancy asthma cases. Increased exposure to yeasts in floor dust and hydrophilic fungi without yeasts in chair dust by IQR significantly increased the odds of being a postoccupancy asthma case by 77% and 44%, respectively.

When we ran models with culturable fungi, ergosterol, endotoxin, and demographic variables simultaneously, the ORs of the respiratory and epi-asthma cases for total fungi and hydrophilic fungi in both floor and chair dust were slightly smaller (IQR-OR = 1.46–1.62 for floor dust and IQR-OR = 1.36–1.57 for chair dust) but generally remained significant at α = 0.05. The exception was the modeling of respiratory cases with total chair fungi (IQR-OR = 1.36; *p* = 0.06) adjusted for ergosterol, endotoxin, and demographic variables (Table 5). In the total fungi models of the multiple environmental variable models, the magnitude of the IQR-ORs for the respiratory and epi-asthma cases associated with total culturable fungi was smaller than that for hydrophilic fungi in the hydrophilic fungi models. Exposure to hydrophilic fungi in floor and chair dust were associated with about a 2-fold increase in the odds of being a post-occupancy asthma case (IQR-OR = 2.09 for floor dust and IQR-OR =1.79 for chair dust) (Table 5). GAMs with nonlinear spline smoothing functions did not provide evidence of nonlinear relationships; that is, the log odds (logit) of respiratory illnesses in all of these multiple environmental variable models increased linearly with increase of exposure. A sensitivity analysis was performed by rerunning all statistical models after assigning floor-specific mean values (new models) of microbial measurements to the respiratory cases and comparisons with no individual measurements. For each exposure variable we calculated the ratio (expressed as percentage) of the OR for the new model to the original model. The results showed that the models were not substantially sensitive because the ORs from the new models ranged from 84.8% to 102.8% of the original ORs presented in Tables 4 and 5.

## Discussion

Among employees of a water-damaged office building, we found linear associations between respiratory illnesses and the levels of fungi, which were largely explained by hydrophilic (water-loving or tertiary) fungi including yeasts. These linear exposure–response relationships based on individual exposure measurements extend our previous findings, which were based on exposure assigned as floor-specific mean values of fungal measurements (Park et al. 2006). The present study indicates that exposure assessment using individual dust samples has an advantage over assigning exposure using floor-specific mean values, because we found associations between physician-diagnosed postoccupancy-onset asthma and fungal exposure that were not demonstrated in the previous study (Park et al. 2006). The enhanced findings are likely to be explained by minimization of exposure misclassification by using individual samples in exposure assessment. Of the three respiratory health outcomes studied, asthma diagnosed after building occupancy had the strongest association with the levels of hydrophilic fungi in dust. This finding implies that a more specific (less sensitive) definition of outcome based on physician diagnosis, which likely represents more severe disease, probably minimized health outcome misclassification in relation to building exposures. We previously reported that the incidence of adult-onset asthma in this population was 7.5 times higher after building occupancy (Cox-Ganser et al. 2005). Taken together, these findings are consistent with the involvement of building-related fungal exposure in the causal chain of adult-onset asthma, although we cannot rule out that fungi—specifically hydrophilic species—may be simply markers of other causative agents in damp environments.

Among hydrophilic fungi, yeasts in both floor and chair dust played an important role in associations we found for increased odds of respiratory illnesses. Yeasts have been reported to be among the most abundant fungi found in indoor air (Cheong et al. 2004; Rantio-Lehtimaki 1988) and in house dust (Flannigan et al. 1993; Verhoeff et al. 1994). Similarly, in the present study, the *Rhodotorula* genus of yeasts was one of the most prevalent and abundant fungi recovered in floor and chair dust. *Rhodotorula* species have been shown to be implicated in IgE-mediated allergy responses (Day and Ellis 2001), as well as being potential causative agents of HP in case studies (Hodges et al. 1974; Siersted and Gravesen 1993). Our study also showed that yeasts in chair dust were significantly associated with an epidemiologic definition of HP (those who reported physician-diagnosed HP or having two or more HP-like symptoms). In occupational environments, such as bakeries, breweries, and distilleries, the yeast *Saccharomyces cerevisiae* is a major allergen source for allergic diseases (Day and Ellis 2001). Without yeasts, the hydrophilic fungi as a group (*Phoma herbarum*, *Chaetomium globosum*, *Mucor plumbeus*, *Rhizopus stolonifer,* and *Stachybotrys chartarum*) were also strongly associated with odds of respiratory illnesses. In these models, we assigned a value of LOD/2 (200 CFU/g) to a large proportion of samples < LOD (Table 2), and these assigned values were multiplied by the amount of dust collected for each subject to obtain CFUs per square meter or per chair. This assignment might have produced nondifferential misclassification in exposure, resulting in the underestimation of ORs. Even with this potential misclassification, we still found significant associations between hydrophilic fungi without yeast and respiratory illnesses. *Phoma* spp., *Mucor* spp., and *Rhizopus* spp. have been implicated in IgE-mediated allergy (Day and Ellis 2001). However, we are not aware of epidemiologic studies demonstrating increased risk of building-related asthma or other respiratory illnesses associated with yeasts and other hydrophilic fungi in floor and chair dusts in water-damaged nonindustrial buildings.

We found poor correlations (*r* < 0.3) between ergosterol levels and culturable fungi in more than 300 dust samples, although Saraf et al. (1997) reported a higher correlation (*r* = 0.65) in 17 house dust samples. This is not a completely unexpected result for the following reasons. First, ergosterol is found in mycelia and fungal fragments, as well as in intact spores (Miller 2001). Second, ergosterol can be detected in both viable and nonviable spores. Third, culturable fungi represent only a small portion of the viable spores that can grow on the selected media (Dales et al. 1999; Saraf et al. 1997). Finally, the proportion of viable and nonviable spores may differ across the samples, and the proportion of the viable spores that can be cultured may also differ. Hence, ergosterol has the potential to measure fungal biomass more accurately than the culture technique. Because health effects such as allergy and inflammation do not rely on viability of fungal contaminants, measuring ergosterol to estimate total fungal biomass in exposure assessment is warranted (Nielsen and Madsen 2000; Pasanen et al. 1999).

A few research groups have used ergosterol measurements for exposure assessment in epi-demiologic studies, but the results have been inconsistent. In a cross-sectional analysis of the European Community Respiratory Health Survey (ECRHS) subcohort followed up in 1996 (*n* = 485), Dharmage et al. (2001) found a significant association of ergosterol levels in bedroom dust with sensitization to fungi and having wheezed in the last year. They also performed a longitudinal analysis on repeated measurements of wheeze and ergosterol in bedroom dust in 1996 and 1998 on the same ECRHS subcohort. They found a statistically significant interaction effect in which the effect of increasing ergosterol on the chance of remission of wheeze depended on the initial levels of ergosterol in 1996 (Matheson et al. 2005). From the same ECRHS subcohort, 35 young asthmatic adults sensitized to fungi were followed over four seasons. No association was found between either culturable fungi or ergosterol levels in bedroom floor dust and peak flow variability. However, Dharmage et al. (2002) discussed that this lack of association could have been partly explained by misclassification of exposure. In a study of children in Canada, Dales (1998) reported a significant association between living in fungal-contaminated homes with higher airborne ergosterol levels and an increased number of CD3+ T cells expressing CD45RO. In a later study, Dales et al. (1999) examined the association of airborne ergosterol levels in bedrooms with respiratory symptoms and nocturnal cough among elementary school children, but they did not find associations. In that study (Dales et al. 1999), airborne ergosterol levels were estimated from less-than-a-day sampling. This might have misclassified children’s exposure in relation to the 1- and 12-month time periods covered by the symptom questionnaire. Mendell et al. (2002) measured airborne ergosterol in a double-blind cross-over study evaluating the effect of replacement of filters on occupants’ symptoms and indoor particles in an office building; they found ergosterol levels < LOD for seven of eight air samples.

In contrast, we found that 230- or 260-ng increases in ergosterol levels per square meter of floor or per chair, respectively, elevated the odds of respiratory illnesses (especially epi-asthma cases) by 46–55% in models adjusted for demographics, culturable fungi, and endotoxin. It is not known whether ergosterol can directly induce respiratory health effects or if it is a surrogate measure of exposure to fungi or of another unmeasured exposure related to dampness. However, our finding of associations between ergosterol and health outcomes, independent of culturable fungi, suggests that measuring both ergosterol and culturable fungi may be important to fully understand health effects associated with fungal exposure in epidemiologic studies.

In the building we studied, which had a long history of water damage, the first major construction activity related to water incursion began in 2000, with repair of roof copings and brick caulking. From 2000 to 2002 cubicle partitions and carpets were cleaned, wetted carpet and stained wallboard replaced, wall-paper and underlying mold removed from bathrooms, upgrades to the air handling system made, and windows caulked. We found that the levels of culturable fungi in dust sampled in 2002, 7 months after the 2001 questionnaire survey, were low compared with those in other studies of office buildings (Chao et al. 2001), school buildings (Ebbehoj et al. 2005), and residential buildings (Hicks et al. 2005) with no apparent water damage. Before our study, the historical levels of fungi were higher. For example, three consultant reports on 20 surface dusts from 2000 to 2001 showed a range of 4,700–7,800,000 CFU/g as compared to our current study range of 276–1,200,000 CFU/g in floor dust (Occupational Risk Control Services, unpublished data). Furthermore, fungal contamination was found in the walls. We surmise that the relative differences in occupant exposure and fungal profile in the dust at individual workstations might have remained even though the remediation action changed the absolute levels of microbial contaminants. This would explain the association between the fungal exposure and health effects even at the low absolute levels of fungi in the dust.

The fungal profile in our dust samples was predominantly hydrophilic and mesophilic fungi. At the time of our study, the carpet and chairs generally showed low *A_w_* (0.18–0.8, with a mean of 0.5). Historical reports indicated that the building had extensive water damage in the past, and our fungal profile analyses support that there must have been wet conditions. Hydrophilic fungi are not likely to become predominant unless wet conditions persist for an extended time (Flannigan and Miller 2001). In a study of houses without water damage, water-indicator fungi (*Chaetomium* spp., *Ulocladium* spp., and *Stachybotrys* spp.) were largely absent from air and dust samples (Horner 2004). Furthermore, because the spores of the four most dominant fungi we found in both floor and chair dusts (yeasts, *Aureobasidium pullulans*, *Alternaria alternata*, and *Epicoccum nigrum*) have long survival times (Flannigan and Miller 2001), they can remain in relatively dry conditions as indicators of past dampness.

Several different fungal genera and species have been used as indicators of water-damaged indoor environments (Burge and Otten 1999; Flannigan and Miller 2001). We know of no source that lists the water activities for all fungi. Furthermore, minimum and optimum *A_w_* for growth of individual fungal species can differ depending on environmental conditions, such as temperature and nutrient availability (Burge and Otten 1999). We categorized mesophilic and hydrophilic fungi based on three reports (Burge and Otten 1999; Flannigan and Miller 2001; Grant et al. 1989), and thus we may have some misclassification. However, because most of the fungi with prevalence of > 10% were classifiable based on those reports, the misclassification in *A_w_* categorization is not likely to change our findings in the study.

In conclusion, we showed that among employees in a building with a long history of water damage, respiratory symptoms and post-occupancy asthma were strongly associated with fungi in a linear exposure–response manner, especially the levels of hydrophilic fungi (including yeasts) in dust. These findings extend the conclusions of insufficient evidence for the development of asthma in relation to the presence of mold or other agents in damp indoor environments reported by the Institute of Medicine (2004). Because the markers (total culturable fungi, hydrophilic fungi, and ergosterol) of potential mold exposure were associated with health outcomes, we suggest that further research to understand respiratory health effects in water-damaged indoor environments include measurements of both ergosterol and speciated culturable fungi in dust.

## Figures and Tables

**Table 1:** Demographics of respiratory cases, epi-asthma cases, current postoccupancy asthma cases, and the comparison group.

Demographics	Total^a^ (*n* = 352)	Respiratory cases (*n* = 200)	Epi-asthma cases (*n* = 123)	Postoccupancy asthma cases (*n* = 49)	Comparison group (*n* = 152)
Age (mean ± SD)	46.4 ± 8.6	46.3 ± 8.4	45.8 ± 7.1	46.4 ± 7.4	46.5 ± 8.8
Race (%)					
White	68.8	65.1	69.8	57.1	73.7
Black	20.7	23.6	20.2	28.6	16.9
Other	10.5	11.3	10.1	14.3	9.5
Sex (% female)	59.3	70.0	66.7	75.5	45.0
Building occupancy in years (mean ± SD)	6.0 ± 1.8	6.1 ± 1.6	6.2 ± 1.5	6.1 ± 1.4	5.9 ± 1.9
Smoking status (%)					
Current	13.1	15.5	17.1	6.1	9.9
Former	25.9	29.0	30.1	38.8	21.7
Never	61.1	55.5	52.9	55.1	68.4

See “Materials and Methods” for definitions of the three case groups and the comparison group.

a Respiratory cases plus comparison subjects.

**Table 2:** Distributions of concentration and load of microbial agents in floor and chair dust.

		Concentration (per g or mg)^a^		Load (per m^2^ floor or per chair)^b^	
Sample type, microbial agent analyzed	< LOD^c^ (*n*/total)	GM (GSD)	IQR	GM (GSD)	IQR
Floor dust					
Total fungi (CFU)	3/328	7,700 (4.7)	14,300	2,000 (5.5)	4,700
Fungi with *A_w_* ≥ 0.8	12/328	4,800 (5.6)	11,100	1,300 (6.6)	3,900
Hydrophilic fungi	53/328	2,600 (7.0)	7,700	700 (7.9)	2,600
Yeasts only	78/328	1,700 (6.7)	5,600	500 (8.1)	1,600
Hydrophilic (no yeasts)	233/328	380 (4.2)	170	100 (4.5)	110
Ergosterol (ng)	0/334	0.5 (3.1)	0.7	126.2 (3.9)	231.8
Endotoxin (EU)	0/338	10.9 (4.1)	28.1	2683.0 (4.8)	7413.6
Cat allergen (μg)	3/314	2.5 (2.4)	2.3	0.7 (2.9)	0.9
Dog allergen (μg)	16/314	2.1 (2.8)	2.6	0.6 (3.2)	1.0
Chair dust					
Total fungi (CFU)	1/326	11,000 (5.4)	31,200	4,900 (5.7)	12,000
Fungi with *A_w_* ≥ 0.8	8/326	7,000 (6.2)	15,700	3,100 (6.6)	7,400
Hydrophilic fungi	56/326	2,600 (8.1)	7,000	1,200 (8.5)	3,800
Yeasts only	118/326	1,100 (6.5)	3,400	500 (7.0)	1,300
Hydrophilic (no yeasts)	181/326	610 (6.7)	600	270 (7.1)	330
Ergosterol (ng)	0/325	0.4 (2.5)	0.5	173.6 (2.9)	260.4
Endotoxin (EU)	0/327	2.1 (2.6)	2.7	932.8 (3.0)	1268.8
Cat allergen (μg)	0/318	12.5 (3.6)	24.8	5.8 (4.1)	9.8
Dog allergen (μg)	4/318	5.7 (3.7)	12.2	2.6 (4.1)	6.5

a Units are ng/mg for ergosterol, EU/mg for endotoxin, CFU/g for fungi, and μg/g for allergens.

b Computed by multiplying the concentration of the microbial agents by the total amount of dust collected in each floor (or chair) sample and then dividing by 2 m^2^ for floor samples.

c LODs are 0.002 EU/mg for endotoxin, 350–400 CFU/g for total culturable fungi depending on the amount of samples analyzed, 0.5 μg/g for cat allergen, and 0.4 μg/g for dog allergen. For samples < LOD, we used LOD/2 for total fungi and 200 CFU/g for the subgroups of total culturable fungi.

**Table 3:** The prevalence and levels of mesophilic and hydrophilic fungal species identified from floor or chair dust samples.^a^

	Floor (*n* = 328)			Chair (*n* = 326)		
	Prevalence No. (%)	Level (CFU/g dust)		Prevalence No. (%)	Level (CFU/g dust)	
Species in samples		Median	Maximum		Median	Maximum
Yeasts^b^	250 (76.2)	2,400	1.2 × 10^6^	208 (63.8)	2,340	8.6 × 10^5^
*Aureobasidium pullulans*	175 (53.4)	770	8.0 × 10^4^	244 (74.8)	1,500	1.5 × 10^6^
*Epicoccum nigrum*	121 (36.9)	400	1.4 × 10^4^	171 (52.4)	710	3.3 × 10^4^
*Alternaria alternata*	70 (21.3)	710	1.1 × 10^4^	74 (22.7)	740	1.8 × 10^4^
*Phoma herbarum*^b^	54 (16.5)	2,850	1.0 × 10^6^	71 (21.8)	3,600	5.9 × 10^6^
*Cladosporium sphaerospermum*	48 (14.6)	1,100	3.0 × 10^4^	51 (15.6)	1,200	1.6 × 10^5^
*Aspergillus niger*	30 (9.1)	390	6.1 × 10^4^	49 (15.0)	380	3.6 × 10^4^
*Chaetomium globosum*^b^	27 (8.2)	770	1.4 × 10^4^	50 (15.3)	390	4.0 × 10^4^
*Cladosporium cladosporioides*	19 (5.8)	400	2.9 × 10^4^	18 (5.5)	1,250	7.7 × 10^3^
*Fusarium solani*	18 (5.5)	400	8.0 × 10^3^	12 (3.7)	710	4.0 × 10^3^
*Cladosporium herbarum*	16 (4.9)	1,800	1.2 × 10^4^	13 (4)	710	2.8 × 10^4^
*Mucor plumbeus*^b^	11 (3.4)	380	3.7 × 10^4^	26 (8.0)	380	4.0 × 10^4^
*Rhizopus stolonifer*^b^	9 (2.7)	370	3.7 × 10^3^	23 (7.1)	370	3.6 × 10^3^
*Ulocladium chartarum*	6 (1.8)	390	3.6 × 10^3^	18 (5.5)	380	7.1 × 10^3^
*Stachybotrys chartarum*^b^	4 (1.2)	2,200	1.7 × 10^4^	5 (1.2)	380	4.0 × 10^3^
*Aspergillus flavus*	2 ( < 1)	2,080	3.8 × 10^3^	2 ( < 1)	2,550	3.7 × 10^3^
*Chrysonilia sitophila*	2 ( < 1)	3,800	3.8 × 10^3^	4 (1.2)	3,700	3.6 × 10^4^
*Penicillium expansum*	2 ( < 1)	1,300	1.5 × 10^3^	ND	ND	ND

ND, not detected.

a The LOD ranged between 350 and 400 CFU/g dust; samples < LOD for individual fungi are not included in statistical analyses presented in this table.

b Hydrophilic fungi; all other species in the table are mesophilic fungi.

**Table 4:** Associations of microbial agents in floor and chair dust with respiratory, epi-asthma, or current postoccupancy asthma cases in single environmental variable models adjusted for age, sex, race, smoking status, and building occupancy time.

	OR (95% CI) for different outcome models					
	Respiratory		Epi-asthma		Postoccupancy asthma	
Environmental variable	Floor dust	Chair dust	Floor dust	Chair dust	Floor dust	Chair dust
Total culturable fungi	1.66** (1.19–2.33)	1.37** (1.02–1.85)	1.72** (1.21–2.46)	1.58** (1.13–2.20)	1.56* (0.96–2.53)	1.67** (1.07–2.60)
*A_w_* ≥ 0.8 fungi^a^	1.66** (1.17–2.38)	1.31** (1.00–1.72)	1.69** (1.15–2.47)	1.46** (1.09–1.97)	1.72** (1.03–2.88)	1.56** (1.05–2.30)
Fungi not classified as *A_w_* ≥ 0.8 fungi	1.11 (0.80–1.54)	1.09 (0.76–1.56)	1.21 (0.84–1.72)	1.11 (0.75–1.64)	0.80 (0.47–1.37)	1.20 (0.67–2.18)
Hydrophilic fungi	1.73** (1.20–2.51)	1.45** (1.07–1.97)	1.80** (1.20–2.69)	1.63** (1.16–2.28)	2.19** (1.23–3.89)	1.85** (1.19–2.89)
Fungi not classified as hydrophilic	1.08 (0.79–1.50)	1.25* (0.96–1.62)	1.12 (0.79–1.59)	1.31* (0.99–1.74)	0.71 (0.42–1.19)	1.31 (0.88–1.96)
Yeasts only	1.37* (0.97–1.93)	1.46** (1.06–2.01)	1.46* (1.00–2.13)	1.42** (1.01–1.99)	1.77** (1.05–3.01)	1.55* (1.00–2.41)
Hydrophilic fungi without yeasts	1.50** (1.18–1.91)	1.21* (0.97–1.51)	1.47** (1.13–1.91)	1.36** (1.06–1.74)	1.43* (0.99–2.05)	1.44** (1.05–1.98)
Ergosterol	1.56** (1.13–2.16)	1.38* (0.98–1.93)	1.60** (1.13–2.28)	1.54** (1.05–2.26)	1.37 (0.87–2.17)	1.63* (0.95–2.81)
Endotoxin	1.60** (1.09–2.37)	1.10 (0.82–1.48)	1.54** (1.01–2.34)	1.09 (0.79–1.52)	1.40 (0.79–2.50)	1.15 (0.71–1.87)
Cat allergen (Fel d 1)	1.33* (0.96–1.83)	1.21 (0.91–1.63)	1.35* (0.95–1.92)	1.37* (1.00–1.88)	1.16 (0.72–1.88)	1.55* (1.00–2.39)
Dog allergen (Can f 1)	1.18 (0.85–1.65)	1.26 (0.86–1.83)	1.09 (0.76–1.57)	1.20 (0.78–1.83)	1.01 (0.62–1.65)	1.10 (0.61–2.00)

ORs and 95% CIs were computed based on change of the IQR in the environmental variable. The number of samples for each model varies from 286 to 303 for respiratory cases, 225 to 241 for epi-asthma cases, and, 170 to 183 for postoccupancy asthma cases. Units of the environmental variables are as follows: CFU/m^2^ for fungi in floor dust and CFU/chair for fungi in chair dust; EU/m^2^ for endotoxin in floor dust and EU/chair for endotoxin in chair dust; ng/m^2^ for ergosterol in floor dust and ng/chair for ergosterol in chair dust; and μg/m^2^ for allergen in floor dust and μg/chair for allergen in chair dust.

a Mesophilic and hydrophilic fungi (Table 3); hydrophilic fungi include yeasts, *Phoma herbarum*, *Chaetomium globosum*, *Mucor plumbeus*, *Rhizopus stolonifer*, and *Stachybotrys chartarum*.

* Statistically significant at α = 0.1.

** Statistically significant at α = 0.05.

**Table 5:** Associations of microbial agents in floor and chair dust with respiratory, epi-asthma, or current postoccupancy asthma cases in multiple environmental variable models adjusted for age, sex, race, smoking status, and building occupancy time.

	OR (95% CI) for different outcome models					
	Respiratory		Epi-asthma		Postoccupancy asthma	
Multiple environmental variable models	Floor dust	Chair dust	Floor dust	Chair dust	Floor dust	Chair dust
Total fungi models						
Total culturable fungi	1.46 (1.02–2.10)**	1.36 (0.99–1.87)*	1.55 (1.05–2.27)**	1.57 (1.09–2.25)**	1.46 (0.88–2.44)	1.60 (0.99–2.58)*
Ergosterol	1.40 (0.97–2.04)*	1.33 (0.93–1.91)	1.54 (1.02–2.34)**	1.46 (0.96–2.23)*	1.22 (0.71–2.11)	1.48 (0.82–2.67)
Endotoxin	1.20 (0.75–1.90)	0.91 (0.65–1.27)	1.07 (0.64–1.80)	0.83 (0.57–1.21)	1.05 (0.53–2.08)	0.87 (0.51–1.48)
Hydrophilic fungi models						
Hydrophilic fungi^a^	1.54 (1.05–2.27)**	1.42 (1.03–1.95)**	1.62 (1.06–2.48)**	1.57 (1.11–2.23)**	2.09 (1.15–3.79)**	1.79 (1.12–2.85)**
Ergosterol	1.41 (0.97–2.05)*	1.32 (0.92–1.89)	1.55 (1.02–2.36)**	1.46 (0.96–2.22)*	1.19 (0.68–2.07)	1.47 (0.81–2.63)
Endotoxin	1.21 (0.76–1.92)	0.93 (0.67–1.28)	1.10 (0.66–1.84)	0.88 (0.61–1.26)	1.02 (0.51–2.05)	0.87 (0.51–1.47)

ORs and 95% CIs were computed based on change of the IQR in the environmental variable for each model. The number of samples for each model varies: 294 or 295 for respiratory cases, 233 or 234 for epi-asthma cases, and 178 or 179 for postoccupancy asthma cases, depending on the amount of dust available for analyses. Priority for the analysis was endotoxin, culturable fungi, ergosterol, and then allergens. Units of the environmental variables are as follows: CFU/m^2^ for fungi in floor dust and CFU/chair for fungi in chair dust; EU/m^2^ for endotoxin in floor dust and EU/chair for endotoxin in chair dust; and ng/m^2^ for ergosterol in floor dust and ng/chair for ergosterol in chair dust.

a Includes yeasts, *Phoma herbarum*, *Chaetomium globosum*, *Mucor plumbeus*, *Rhizopus stolonifer*, and *Stachybotrys chartarum*.

* Statistically significant at α = 0.1.

** Statistically significant at α = 0.05.
